# Supplementary material for: Cognition and Behavior of COVID-19 Vaccination Based on the Health Belief Model: A Cross-Sectional Study
Source: Vaccines (Basel). 2022 Apr 1;10(4):544. doi: 10.3390/vaccines10040544 (PMC9030847; doi:10.3390/vaccines10040544)
Supplement: Supplementary file 1 [file vaccines-10-00544-s001.zip › vaccines-1634290-supplementary.pdf]

Supplementary materials

Table S1. Items Based on HBM Constructs.

HBM Constructs	Items
Perceived susceptibility	1.How likely do you think you will get COVID-19 in the coming 12 months?<br>2.I believe that if I do not get vaccinated, the likelihood of me getting infected with corona will increase.<br>3.I believe that if I do not get vaccinated, the likelihood of my family and relatives getting infected in Corona will increase.
Perceived severity	1.If you get COVID-19, how serious do you think the consequences will be?<br>2.What is the possibility of having long-term health or economic sequelae if getting COVID-19?<br>3.If children/the elderly get COVID-19, how serious do you think the consequences will be?
Perceived benefits	1. Do you think COVID-19 vaccine can reduce the chance of infection?<br>2. Do you think COVID-19 vaccine can decrease the severity and the chance of having complications if people are infected?<br>3. Do you think COVID-19 vaccine can prevent people from spreading the virus to others?
Perceived barriers	1. The COVID-19 vaccine causes a person to get COVID-19.<br>2. People will have serious sequelae after getting the COVID-19 vaccine.<br>3. Getting vaccinated requires time and effort.
Cues to action	1. The chances of me getting vaccinated against COVID-19 will increase if opinion leaders on social media express support for the benefit of the vaccine.<br>2. The chances of me getting vaccinated against COVID-19 will increase if friends and family express support for the benefit of the vaccine.<br>3. The chances of me getting vaccinated against COVID-19 will increase if official guidelines from the Ministry of Health are published.<br>4. The chances of me getting vaccinated against COVID-19 will increase if my GP recommends me.<br>5. If my workplace takes care of vaccinating the workers against COVID-19, I will vaccinate.
Health Behavior	1. I exercise as recommended for my age.<br>2. I make sure to eat a healthy and varied diet.<br>3. I have a good sleep habit.

Score each of the items from 1 (completely disagree) to 5 (agree completely).
